# GCMR-IMA: graph-based cross-modal retrieval with incomplete modality awareness

**DOI:** 10.1038/s41598-026-58071-3

**Published:** 2026-06-23

**Authors:** Rui Wang, Xianghong Tang, Jianguang Lu

**Affiliations:** State Key Laboratory of Public Big Data, Guizhou University, Guiyang, Guizhou 550025 China

**Keywords:** Cross-modal retrieval, Incomplete modality awareness, Semantic graph learning, Dual-embedding architecture, Image-text retrieval, Computational biology and bioinformatics, Health care, Mathematics and computing

## Abstract

Cross-modal retrieval, especially for image-text pairs, plays a vital role in areas such as medical image analysis, multimedia search, and personalized recommendations. However, challenges like modality heterogeneity, missing data, and noise still limit its effectiveness. Traditional methods often suffer from high computational complexity, while deep learning-based approaches struggle to capture long-range semantic relationships and handle missing modalities. To address these issues, we propose a graph-based Incomplete Modality Awareness Cross-Modal Retrieval method (GCMR-IMA) with four key innovations: (1) A dual-layer semantic architecture with image-text dual embedding for global semantic graph learning and long-range semantic capture; (2) A sparsified modality adjacency graph to model latent inter-modality relationships, improving modality association awareness; (3) A graph-guided missing modality awareness method, providing semantically consistent guidance for missing modalities using the global semantic graph’s sparsified adjacency matrix; (4) An adaptive loss function that optimizes modality projections for context-aware missing modality detection. Extensive experiments demonstrate that GCMR-IMA continues to perform well in the presence of missing modalities. This framework enhances the robustness and effectiveness of cross-modal retrieval systems, supporting better decision-making in real-world applications.

## Introduction

Modal retrieval, which refers to retrieving information from various modalities such as text, images, and audio, has become an essential research topic in the era of big data and multimodal information systems. With the explosive growth of multimodal data across domains like medical image analysis, social media, and personalized recommendation systems, effectively integrating and retrieving information across different modalities has gained increasing importance. However, traditional retrieval methods often struggle to capture complex semantic relationships between heterogeneous modalities, leading to issues such as semantic gaps, insufficient alignment, and degraded retrieval accuracy. Cross-modal retrieval has emerged to address these challenges by learning shared representations that bridge different modalities and enable efficient retrieval across them–for example, retrieving a textual report given an image or vice versa. Solving these challenges not only enhances retrieval performance and efficiency but also contributes to broader applications such as intelligent diagnosis, content understanding, and human–computer interaction, thereby offering significant practical and scientific value. Cross-modal retrieval methods are generally categorized into two main approaches: traditional methods and deep learning-based methods.

Early traditional methods relied on hand-crafted features for modality mapping. These involved extracting low-dimensional features from each modality and matching them for retrieval. For example, canonical correlation analysis (CCA) has been shown to improve retrieval accuracy by modeling correlations between components^1^. Similarly, kernel canonical correlation analysis (KCCA) has been used to learn semantic representations for web images and associated text^2^. Other approaches, such as joint feature selection, map multi-modal data into a common subspace to measure similarity^3^. While these methods have proven reliable, they struggle with large-scale multi-modal data due to high computational complexity, distribution shifts, and maintaining semantic consistency. To address this, cross-modal hashing methods based on collective matrix factorization have been proposed^4^, improving matrix factorization’s discriminative power by incorporating label and geometric consistency. However, challenges with hash encoding quality and computational efficiency remain.

With the rise of deep learning, neural network-based cross-modal retrieval has become more prevalent, offering automatic feature space mapping. Early deep learning approaches focused on feature representation, using convolutional neural networks (CNNs)^5^ as feature extraction backbones. These methods map images and texts into a shared space to learn modality associations, often using cosine similarity as a distance metric, with promising results^6^. However, these methods are limited by their focus on vector direction and inability to capture local feature information. Approaches like Corr-AE^7^ combine autoencoders with error minimization between modality representations, achieving good results on several public datasets. Deep-SM^8^ integrates pre-trained CNNs with deep semantic matching to address cross-modal retrieval with multiple labels. However, these methods face challenges in fully aligning semantics due to significant distribution differences between modalities.It requires learning a shared representation space for matching data from multiple modalities. However, challenges such as modality heterogeneity, missing data, and noise complicate the processes of these methods.

Recent works focusing on modality-invariant representations have gained attention. DSCMR^9^ learns modality-invariant features by minimizing modality invariance loss. C3CMR^10^ enhances the discriminative power of single-modal features based on intra-modality similarity and incorporates multi-level, class-level dual losses to learn cross-semantic associations. However, these methods overlook the loss of specific information during the modality-invariant representation process, which affects fine-grained semantic alignment. To address specific challenges, recent research combines various techniques. For example, DAVAE^11^ employs multiple autoencoders to learn latent factors between modalities, thereby achieving dual alignment at the semantic level of distribution, and utilizes generative models to synthesize latent representations for missing modalities. Given the entanglement between latent representations and semantically irrelevant features, SDCMR^12^ decouples modality representations and reconstructs features by exchanging shared semantics, ensuring semantic consistency. However, due to optimization conflicts, overfitting, and nonlinear coupling, these methods require further exploration to strike a balance between modality completion and hierarchical feature modeling. In addition, studies inspired by graph neural networks (GNNs)^13^ have also emerged. For instance, DGMS^14^ captures deep semantic relationships between modalities using bilingual semantic graphs and multiple subspaces, applying dual learning to obtain joint probability distributions for modalities. ICMR-DCT^15^ employs graph attention mechanisms in autoencoders to constrain dual cross-modal alignment and project multi-modal representations into a shared semantic space. However, these methods face challenges such as semantic fragmentation and static graph structures, which lack dynamic methods for capturing globally consistent semantics. Standard GNNs generally assume that the node features used to estimate graph topology are reliable and complete. When one modality is unavailable, directly inserting zero or random placeholders may distort pairwise similarities and introduce erroneous edges, while repeated neighborhood aggregation can further propagate missing or noisy signals. Moreover, standard GNNs aggregate observed information but do not explicitly generate a retrievable representation for the absent modality. Therefore, the key challenge is to provide incomplete samples with a usable semantic anchor before graph construction and then exploit information from semantically related samples through constrained graph propagation and explicit missing-modality generation.

To address the aforementioned issues, we propose a graph-based method for incomplete modality-aware cross-modal retrieval (GCMR-IMA) for image-text pairs. In terms of feature architecture, GCMR-IMA employs a dual embedding approach for image-text pairs, enabling the learning of global semantic graphs and capturing long-range semantics. For generating missing modalities, GCMR-IMA utilizes the sparsified adjacency matrix of the global semantic graph to capture latent relationships between modalities. Unlike sample-wise generation that mainly relies on the available counterpart of the same sample, the graph structure enables incomplete samples to aggregate transferable information from semantically related neighbors. In summary, our contributions are as follows:

- We propose GCMR-IMA, a novel end-to-end cross-modal retrieval framework that introduces a dual-layer semantic architecture guided by a modality adjacency graph. This framework establishes a unified structure for modeling hierarchical semantic interactions and adaptive modality dependencies, providing a flexible and robust foundation for image-text retrieval tasks under missing-modality conditions.
- The proposed framework integrates modality dual embeddings with global semantic graph dependencies to form a dual-layer feature architecture, which enhances hierarchical spatial–semantic topology modeling and improves the perception of sparsified modality associations.
- To handle missing modalities, GCMR-IMA introduces a graph-guided missing-modality awareness mechanism, which first assigns a lightweight modality-aware initialization to unavailable modality slots and then generates semantically consistent representations through a sparsified adjacency matrix and a learnable weight-based projection process.
- Extensive experiments conducted on multiple benchmark datasets demonstrate that GCMR-IMA achieves superior performance compared with state-of-the-art methods, confirming its strong capability for robust and context-aware cross-modal retrieval.

## Related work

In this section, we review cross-modal retrieval, focusing on two key areas: unimodal retrieval and multimodal retrieval. We also explore the technological evolution in these domains.

### Unimodal retrieval

Unimodal retrieval, which involves information retrieval within a single modality (e.g. text, image, or audio), has been extensively studied. This technique calculates similarity between queries and candidate data within the same modality, avoiding the “heterogeneity gap” encountered in cross-modal retrieval. Unimodal retrieval can be categorized into text retrieval^16^ and image retrieval^17^, among others. Text retrieval is further divided into sparse^18^ and dense approaches^19^. Sparse retrieval typically relies on keyword-matching statistical models. For example, TF-IDF^20^ computes relevance based on term frequency-inverse document frequency, while BM25^21^ improves retrieval efficiency and cross-domain adaptability using a probabilistic ranking model. In contrast, dense retrieval uses deep learning models–such as BERT^22^, RoBERTa^23^, and other Transformer-based^24^ architectures–to map texts into low-dimensional embeddings for semantic matching. Notably, ColBERT^25^ introduces late interaction mechanisms, greatly enhancing fine-grained matching performance. Despite their maturity, these methods still struggle with comprehending complex semantics.

### Multimodal retrieval

Significant progress has been made in multimodal retrieval research in recent years^26^. Unlike unimodal retrieval, multimodal retrieval improves the comprehensiveness and accuracy of the retrieval process by integrating various data types, such as text, images, and audio, using cross-modal semantic alignment and fusion techniques^27^.

Multimodal retrieval research can generally be categorized into three areas: representation learning, association capturing, and fusion strategies. In representation learning, CCA^2^ aligns network images and their textual semantics using kernel canonical correlation analysis for inter-modal consistency learning. In contrast, Corr-AE^7^ learns hidden layer representations of text-image pairs through a linear combination of joint modality autoencoder errors, achieving constraint alignment at the feature level. Deep-SM^8^ is pretrained on large-scale datasets to obtain a general image representation through CNNs, and, combined with deep semantic matching, addresses the cross-modal retrieval problem in multi-label samples. C3CMR^10^ uses intra-modal similarity to learn cross-semantic associations and inter-modal invariance. In association capturing, CMDN^28^ captures inter-modal complementarity via hierarchical learning and supports deep strategy networks combining intra- and inter-modal representations. The adversarial ACMR^29^ achieves complementary fusion through feature projection and adversarial learning of modality classification. Unlike ACMR, DDAC^30^ minimizes the feature projection distance and fuses label information to eliminate synonymy and heterogeneity across modalities. Additionally, AAL^31^ combines adaptive adversarial methods in its loss function, balancing contributions based on the degree of discreteness in the data. In joint embedding space capturing, DSCMR^9^ minimizes the discriminative loss in both the label and shared representation spaces to guide the model in learning discriminative features. Regarding modality fusion, inspired by GNNs, DGMS^14^ constructs a dual-semantic graph to represent the deep semantics of modality data, capturing both relevance and divergence of modality data through the multi-subspace dual-graph structure. To address the entanglement of semantically irrelevant features across modalities, SDCMR^12^ decouples shared semantics from entangled features using a variational autoencoder, employing a dual-adversarial mechanism to ensure semantic consistency in feature learning.

While many of these approaches focus on complete modality combinations, research on learning missing modalities remains limited. To address this, DAVAE^11^ introduces a dual-alignment variational autoencoder for incomplete cross-modal retrieval (CMR). DAVAE performs dual alignment based on latent factors between different modalities to reduce modality gaps and improve representation discriminability. However, most modalities exhibit spatial dependencies; for example, text descriptions naturally include positional relationships, such as “a bird flying over the water” or “a dog running on the ground,” which align semantically with corresponding image modalities. DAVAE, however, overlooks the joint topological dependency learning between modalities, leaving room for improvement in effective hierarchical spatial topological feature modeling. These limitations motivate a graph-based formulation that combines reliable topology construction, constrained message propagation, and explicit missing-modality generation, rather than treating each incomplete sample independently. Recent graph representation learning studies further support the need for cautious and structured relational modeling. Yan et al.^32^ study graph anomaly detection at critical crossroads, highlighting that structurally important graph regions require careful treatment because unreliable messages may strongly influence downstream representations. Liu et al.^33^ investigate dictionary-based multi-modal temporal graph learning, showing the relevance of structured graph modeling for multi-modal and temporal dependencies. Different from these works, GCMR-IMA focuses on incomplete image–text retrieval, where the central problem is to construct a usable semantic graph under missing-modality conditions and then use it to guide missing-modality-aware retrieval.

## Methodology

**Fig. 1:**
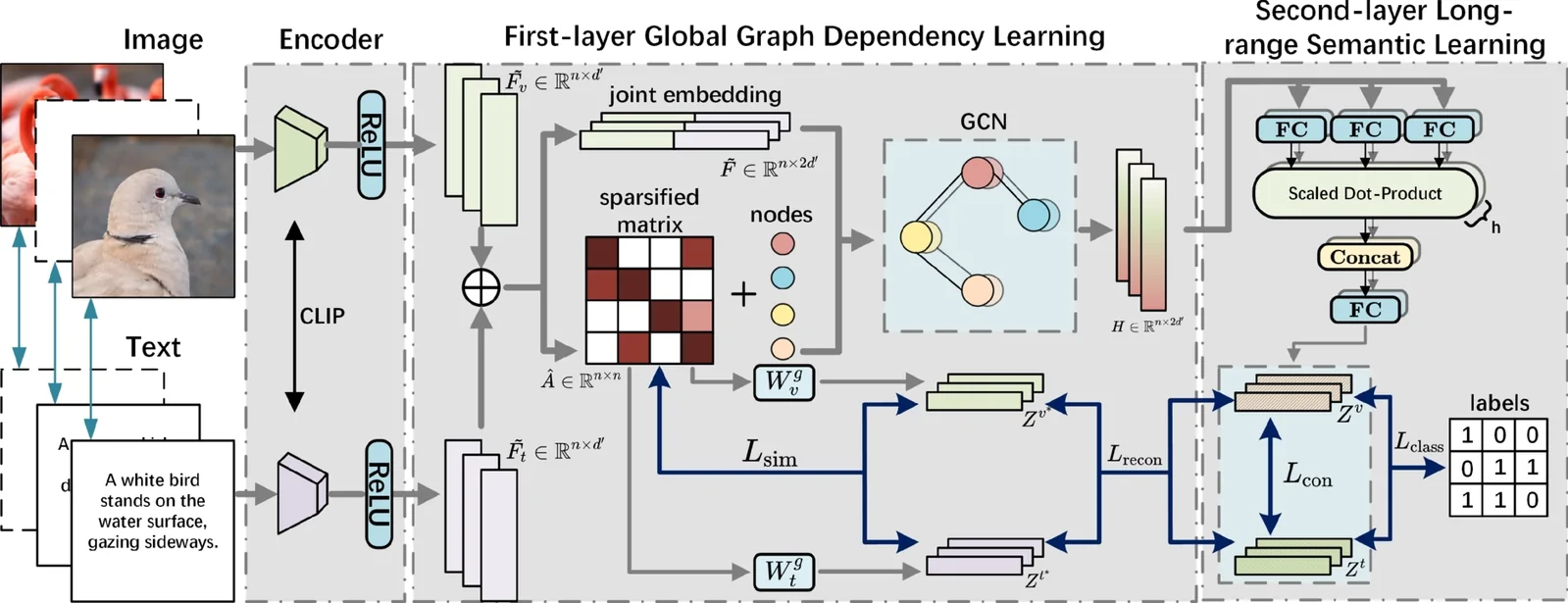
The architecture diagram of GCMR-IMA, where FC denotes a fully connected layer and *⊕* represents vector concatenation.

In this section, we first define the incomplete cross-modal retrieval task and utilize a CLIP^34^ encoder to extract unified image and text features. These features are then projected into a shared semantic space, where a *cross-modal semantic graph* is constructed to capture structural correlations. We apply *global graph dependency learning* via graph convolutional network (GCN)^35^ and enhance it with *multi-head attention* to model long-range semantic relations. To address missing modalities, we introduce an *incomplete modality generation method* that infers absent modality representations based on learned cross-modal associations. Finally, a *composite loss* integrates contrastive, classification, similarity, and reconstruction objectives to guide the unified learning process, as shown in Fig. 1.

### Problem formulation

In fully cross-modal retrieval, given a bimodal dataset $\mathfrak {D}$ with *n* samples:

1 $$\begin{aligned} \mathfrak {D} = (V, T, Y) \end{aligned}$$

where $V = {v_i}{i=1}^n \in \mathbb {R}^{n \times d_v}$ denotes the image set, and $T = {t_i}{i=1}^n \in \mathbb {R}^{n \times d_t}$ represents the corresponding textual descriptions. Here, $d_v$ and $d_t$ are the feature dimensions of images and texts, respectively. The associated label set is $Y = {y_i}_{i=1}^n \in {0,1}^{n \times c}$, where *c* is the number of modality categories.

In incomplete cross-modal retrieval, the image set is defined as $V = {v_i}{i=1}^{n_v} \in \mathbb {R}^{n_v \times d_v}$, and the text set as $T = {t_i}{i=1}^{n_t} \in \mathbb {R}^{n_t \times d_t}$, where $n_v$ and $n_t$ denote the numbers of unimodal image and text samples, respectively, and $n_p$ represents the number of paired image-text samples. In this setting, incomplete cross-modal retrieval addresses the challenge of missing modalities, where training and retrieval are performed with partial or incomplete data, unlike the fully cross-modal setting, which requires complete data from all modalities.

### Image-text encoding

To streamline the model training process, GCMR-IMA employs the CLIP encoder to extract features from raw image and text data. These features serve as the input representations for subsequent modules. CLIP is a multimodal pre-trained model based on contrastive learning, aiming to embed both images and texts into a shared high-dimensional (1024-dimensional) semantic space. This alignment ensures that semantically consistent image-text pairs are closely positioned in the feature space, as expressed by:

2 $$\begin{aligned} F_v&= f_{\text {clip}}(V, \theta _{\text {clip}}) \end{aligned}$$

3 $$\begin{aligned} F_t&= f_{\text {clip}}(T, \theta _{\text {clip}}), \end{aligned}$$

where $\theta _{\text {clip}}$ denotes the parameters of the CLIP model, and $F_v$ and $F_t$ are the *d*-dimensional image and text embeddings, respectively.

To encourage alignment between matched image-text pairs while pushing apart mismatched ones, CLIP employs a symmetric contrastive loss with temperature scaling:

4 $$\begin{aligned} \mathcal {L}{\text {clip}}&= \frac{1}{2n} \sum _{i=1}^{n} \left[ -\log \frac{\exp (\text {sim}(\hat{e}_v^i, \hat{e}_t^i)/\tau )}{\sum _{j=1}^{n} \exp (\text {sim}(\hat{e}_v^i, \hat{e}_t^j)/\tau )} - \log \frac{\exp (\text {sim}(\hat{e}_t^i, \hat{e}_v^i)/\tau )}{\sum _{j=1}^{n} \exp (\text {sim}(\hat{e}_t^i, \hat{e}_v^j)/\tau )} \right] , \end{aligned}$$

where $\text {sim}(\cdot , \cdot )$ denotes the cosine similarity, defined as $\text {sim}(x, y) = \frac{x^\top y}{|x||y|}$, and *τ* is a learnable temperature parameter controlling the distribution sharpness. The embeddings $\hat{e}_v^i$ and $\hat{e}_t^i$ are the $\ell _2$-normalized features of the *i*-th image and text:

5 $$\begin{aligned} \hat{e}_v^i = \frac{F_v^i}{|F_v^i|}, \quad \hat{e}_t^i = \frac{F_t^i}{|F_t^i|}. \end{aligned}$$

This contrastive loss ensures that matched image-text pairs $(\hat{f}_v^i, \hat{f}_t^i)$ are encouraged to be similar, while unmatched pairs $(\hat{f}_v^i, \hat{f}t^j), j \ne i$ are pushed apart in the semantic space. Although the CLIP model is frozen during our training process, the contrastive loss $\mathcal {L} _ {\text {clip}}$ applied during CLIP’s pre-training guarantees that the extracted image and text embeddings are semantically aligned in a shared embedding space. This pre-alignment facilitates effective cross-modal matching, even when modalities are missing, and provides a robust foundation for subsequent hierarchical semantic learning.

### Cross-modal hierarchical semantic learning

Both images and texts exhibit rich structural semantics. Images offer spatial structure and object-level patterns, while texts convey complementary, abstract, and hierarchical descriptions. To fully exploit these heterogeneous yet correlated modalities, GCMR-IMA performs unified hierarchical semantic modeling over the CLIP-extracted features, enabling deeper cross-modal interaction and alignment.

Given the encoded features $F_v, F_t \in \mathbb {R}^{n \times d}$, we first project them into a shared semantic space via independent linear mappings:

6 $$\begin{aligned} \tilde{F}_v&= \text {Dropout}(\text {ReLU}(W_v F_v + b_v)) \end{aligned}$$

7 $$\begin{aligned} \tilde{F}_t&= \text {Dropout}(\text {ReLU}(W_t F_t + b_t)), \end{aligned}$$

where $W_v, W_t \in \mathbb {R}^{d' \times d}$ and $b_v, b_t \in \mathbb {R}^{d'}$ are learnable parameters. The resulting $\tilde{F}_v, \tilde{F}_t \in \mathbb {R}^{n \times d'}$ are embedded into a unified *d'*-dimensional space for structural modeling.

In addition, for missing modalities, we adopt a lightweight preprocessing strategy before semantic graph construction so that both complete and incomplete samples can be represented in a unified form. For the *i*-th sample, let $m_i^v,m_i^t\in \{0,1\}$ denote the availability indicators of the visual and textual modalities, respectively, where at least one modality is observed. The graph-construction embeddings are defined as

8 $$\begin{aligned} \bar{F}_{v,i}&=m_i^v\tilde{F}_{v,i} +(1-m_i^v)\phi _{t\rightarrow v}(\tilde{F}_{t,i}), \end{aligned}$$

9 $$\begin{aligned} \bar{F}_{t,i}&=m_i^t\tilde{F}_{t,i} +(1-m_i^t)\phi _{v\rightarrow t}(\tilde{F}_{v,i}), \end{aligned}$$

where the cross-modal preprocessing functions are

10 $$\begin{aligned} \phi _{t\rightarrow v}(\tilde{F}_{t,i})&=\operatorname {LayerNorm}\!\left( \tilde{F}_{t,i}P_{t\rightarrow v}+e_v^{\textrm{miss}} \right), \end{aligned}$$

11 $$\begin{aligned} \phi _{v\rightarrow t}(\tilde{F}_{v,i})&=\operatorname {LayerNorm}\!\left( \tilde{F}_{v,i}P_{v\rightarrow t}+e_t^{\textrm{miss}} \right). \end{aligned}$$

Here, $P_{t\rightarrow v},P_{v\rightarrow t}\in \mathbb {R}^{d'\times d'}$ are learnable preprocessing matrices, while $e_v^{\textrm{miss}},e_t^{\textrm{miss}}\in \mathbb {R}^{d'}$ are learnable missing-modality prompt embeddings. For an observed modality, the corresponding projected CLIP feature is retained unchanged; for an unavailable modality, a proxy embedding is initialized from the available modality. This proxy embedding is used only to provide a stable representation for graph construction and is subsequently refined by graph propagation and the missing-modality generation module.

The resulting complete node representation is

12 $$\begin{aligned} \bar{F}_i&=\left[ \bar{F}_{v,i};\bar{F}_{t,i}\right] , \nonumber \\ \bar{F}&=\left[ \bar{F}_v;\bar{F}_t\right]. \in \mathbb {R}^{n\times 2d'}. \end{aligned}$$

To capture higher-order semantic dependencies across both modalities, we construct a cross-modal semantic graph from $\bar{F}$ based on cosine similarity:

13 $$\begin{aligned} S_{ij} = \frac{(\bar{F}^{(i)})^\top \bar{F}^{(j)}}{\Vert \bar{F}^{(i)}\Vert \Vert \bar{F}^{(j)}\Vert }, \quad i,j = 1, \dots , n, \end{aligned}$$

where $S \in \mathbb {R}^{n \times n}$ encodes the semantic proximity among all complete and incomplete samples after missing-aware preprocessing, and $\bar{F}^{(i)}$ denotes the joint embedding of the *i*-th sample. We retain the top-*k* neighbors per node to obtain the sparse adjacency matrix $A \in \mathbb {R}^{n \times n}$.

To model global dependencies across modalities, GCMR-IMA applies a GCN on the shared graph. This allows the propagation of both local and non-local semantic cues, enhancing cross-modal feature learning:

14 $$\begin{aligned} \hat{A}&= A + I \end{aligned}$$

15 $$\begin{aligned} H&= \sigma \left( \hat{D}^{-\frac{1}{2}} \hat{A} \hat{D}^{-\frac{1}{2}} \bar{F} W_{\text {gcn}} \right) , \end{aligned}$$

where $\hat{A}$ (adjacency matrix with self-loops) and $\hat{D}$ (degree matrix) govern graph propagation, while $W_{\text {gcn}} \in \mathbb {R}^{2d' \times 2d'}$ projects features to the GCN’s output. After activation $\sigma (\cdot )$, the output $H \in \mathbb {R}^{n \times 2d'}$ integrates cross-modal features through global aggregation.

To further capture long-range semantic dependencies and enhance global contextual reasoning, we apply a multi-head attention mechanism atop the GCN outputs. Specifically, we treat the output *H* as both the query and the key-value source, and compute attention as follows:

16 $$\begin{aligned} \text {Attention}(Q, K, V)&= \text {softmax} \left( \frac{QK^\top }{\sqrt{d_k}} \right) V, \end{aligned}$$

where $Q = H W^Q$, $K = H W^K$, $V = H W^V$, and $W^Q, W^K, W^V \in \mathbb {R}^{d' \times d_k}$ are learnable projection matrices. To enhance representation diversity, *m* parallel attention heads are employed and concatenated:

17 $$\begin{aligned} Z&= \text {Concat}(\text {head}_1, \dots , \text {head}_m) W^O, \end{aligned}$$

where each $\text {head}_i = \text {Attention}(Q_i, K_i, V_i)$ is computed with independent projections, and $W^O \in \mathbb {R}^{m\cdot d_k \times d'}$ is the final output projection matrix. The resulting $Z \in \mathbb {R}^{n \times 2d'}$ captures multi-granular alignment and contextual cues across modalities.

Finally, we separate *Z* into image-specific and text-specific embeddings:

18 $$\begin{aligned} Z^v = Z_{1:d'}, \quad Z^t = Z_{d':2d'}, \end{aligned}$$

where $Z^v, Z^t \in \mathbb {R}^{n \times d'}$ serve as the enhanced hierarchical representations for image and text, respectively. These semantically enriched features are subsequently used in the contrastive objective to facilitate cross-modal retrieval.

### Incomplete modality feature generation

A key challenge in incomplete cross-modal retrieval is generating features for samples with missing modalities. For instance, when images lack corresponding textual descriptions or texts lack paired images, accurate retrieval must still be achieved. It requires robust semantic inference beyond paired supervision. To address this, GCMR-IMA leverages semantic transformation to reconstruct the missing modality by utilizing both explicit and implicit cross-modal associations. Explicit associations arise from shared category labels, providing coarse semantic constraints. In contrast, implicit associations capture the latent structural alignment between visual and textual modalities, as they often describe the same entity in different forms. Formally, GCMR-IMA reuses the semantic graph $\hat{A} \in \mathbb {R}^{n \times n}$, constructed in the hierarchical semantic module, to propagate semantic context from the available modality. Given the missing-aware image and text embeddings $\bar{F}_t$ and $\bar{F}_v$, the missing modality embeddings are further refined as:

19 $$\begin{aligned} Z^{v^*}&= \hat{A} W_{t\rightarrow v}^{\textrm{gen}} \bar{F}_t, \nonumber \\ Z^{t^*}&= \hat{A} W_{v\rightarrow t}^{\textrm{gen}} \bar{F}_v, \end{aligned}$$

where $W_{t\rightarrow v}^{\textrm{gen}}, W_{v\rightarrow t}^{\textrm{gen}} \in \mathbb {R}^{n \times n}$ are trainable sample-wise transformation matrices for the text-to-vision and vision-to-text pathways, respectively. The adjacency matrix $\hat{A}$ performs graph propagation, while $W_{t\rightarrow v}^{\textrm{gen}}$ and $W_{v\rightarrow t}^{\textrm{gen}}$ adapt the sample-wise semantic dependencies. $Z^{v^*}$ and $Z^{t^*}$ are the generated features for the missing vision and text modalities, respectively.

This process allows semantic signals from available modalities to guide the synthesis of missing features, ensuring alignment in the joint embedding space:

20 $$\begin{aligned} Z^{(*)} \in \mathbb {R}^{n \times d'}, \quad \text {where} \quad Z^{(*)} \approx Z^{v} \ \text {or} \ Z^{t}. \end{aligned}$$

By leveraging both the learned semantic graph structure and task-specific transformations, GCMR-IMA enables effective feature generation under asymmetric input conditions, thereby extending retrieval capability beyond fully paired data.

### Loss function design

To learn modality-invariant shared representations and constrain the generation of missing modality features, GCMR-IMA incorporates a composite loss function composed of classification, contrastive, similarity, and reconstruction losses. Each component targets a distinct optimization objective, collectively enhancing the model’s cross-modal learning capacity.

Classification loss ensures that predictions from different modalities align with ground-truth labels, improving discriminative capability. This loss supervises the outputs of both visual and textual classification heads to approximate the true label distribution:

21 $$\begin{aligned} L_{\text {class}} = \frac{1}{n_p} \sum _{i=1}^{n_p} \left( (Y_i^v - Y_i) + (Y_i^t - Y_i) \right) , \end{aligned}$$

where $Y^v$ and $Y^t$ denote the predicted class distributions for the visual and textual modalities, respectively, generated by a shared classification function $f_{\text {class}}$.

Contrastive loss aims to reduce the representational discrepancy of paired samples across modalities, thereby promoting cross-modal alignment. By encouraging embeddings from different modalities to converge within a shared space, this loss enhances retrieval and matching performance:

22 $$\begin{aligned} L_{\text {con}} = \frac{1}{n_p} \sum _{i=1}^{n_p} \left\| Z_i^v - Z_i^t \right\| . \end{aligned}$$

Similarity loss constrains the semantic relationships among generated modality features to approximate the structure of the original modality’s similarity matrix *A*. This ensures that the inter-sample relationships within generated features remain consistent with the semantic topology of the true modality:

23 $$\begin{aligned} L_{\text {sim}} = \left\| Z^{v^*} Z^{v^*T} - \hat{A} \right\| _F^2 + \left\| Z^{t^*} Z^{t^*T} - \hat{A} \right\| _F^2, \end{aligned}$$

where $\Vert \cdot \Vert _F$ denotes the Frobenius norm.

Reconstruction loss minimizes the cosine distance between generated and original features, ensuring high-fidelity synthesis of the missing modality. It drives the generated representations toward the true modality distributions:

24 $$\begin{aligned} L_{\text {recon}} = \frac{1}{n^v} \sum _{i=1}^{n^v} \left( 1 - \frac{Z_i^v {Z_i^{v^*}}^\top }{\Vert Z_i^v\Vert \Vert Z_i^{v^*}\Vert } \right) + \frac{1}{n^t} \sum _{i=1}^{n^t} \left( 1 - \frac{Z_i^t {Z_i^{t^*}}^\top }{\Vert Z_i^t\Vert \Vert Z_i^{t^*}\Vert } \right) . \end{aligned}$$

To optimize for more discriminative, robust, and consistent shared cross-modal representations, we adopt uncertainty-based loss weighting instead of directly learning the unconstrained coefficients. Let $\mathcal {R}=\{\textrm{con},\textrm{class},\textrm{sim},\textrm{recon}\}$ and let $s_r=\log \sigma _r^2$ denote the learnable log-variance parameter of the *r*-th loss. The unified loss is defined as:

25 $$\begin{aligned} L = \sum _{r \in \mathcal {R}} \left( \exp (-s_r)L_r + s_r \right) , \end{aligned}$$

where the effective weight $\exp (-s_r)$ is always positive. The additive term $s_r$ discourages a trivial solution in which a loss is suppressed by driving its effective weight toward zero. The gradient with respect to each log-variance parameter is

26 $$\begin{aligned} \frac{\partial L}{\partial s_r} = 1 - \exp (-s_r)L_r. \end{aligned}$$

All $s_r$ are initialized to zero and jointly updated with the network parameters by the same optimizer through backpropagation, without additional non-negative constraints, normalization, or hard clipping .

The integration of these losses provides a comprehensive optimization objective that simultaneously tackles the challenges of modality absence, semantic divergence, and generation consistency, as formally outlined in Algorithm 1.

**Algorithm 1:**
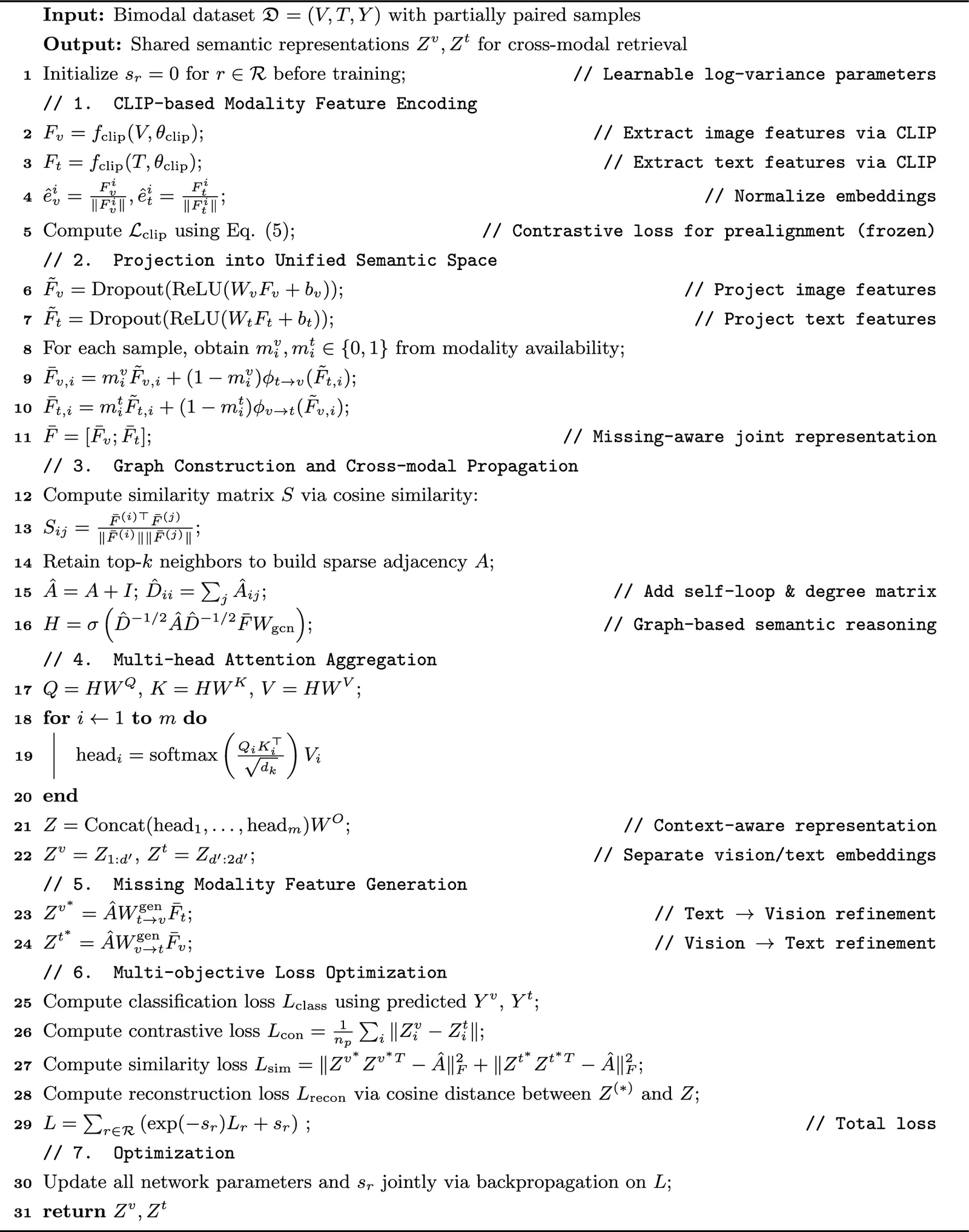
The algorithmic framework of GCMR-IMA.

### Computational complexity

Let *n* denote the mini-batch size, $d_v$ and $d_t$ the input feature dimensions, *d'* the shared embedding dimension, *k* the number of retained neighbors, *c* the number of categories, and $n_p$ the number of paired samples in a mini-batch. Since the CLIP encoder is frozen, its features can be extracted offline and its forward cost is excluded from the trainable complexity. Table 1 summarizes the complexity of each trainable module.

**Table 1:** Time complexity of each module in GCMR-IMA.

Module	Time complexity
Feature projection	$O\!\left( n(d_v+d_t)d'\right)$
Modality-aware initialization	$O(nd'^2)$
Semantic graph construction	$O\!\left( n^2d'+n^2\log k\right)$
GCN propagation	$O\!\left( nkd'+nd'^2\right)$
Multi-head attention	$O\!\left( n^2d'+nd'^2\right)$
Missing-modality generation	$O\!\left( n^2d'+nkd'\right)$
Composite loss	$O\!\left( n^2d'+nc+n_pd'\right)$

The dominant costs arise from graph construction, multi-head attention, and the dense sample-wise transformation in Eq. (15), all of which contain $O(n^2d')$ terms. Top-*k* sparsification reduces GCN propagation from dense $O(n^2d')$ to *O(nkd')*. Therefore, the overall trainable complexity is

27 $$\begin{aligned} O\!\left( n(d_v+d_t)d' +n^2d' +nd'^2 +nkd' +n^2\log k +nc +n_pd' \right) , \end{aligned}$$

which is dominated by $O(n^2d'+nd'^2)$. Relative to the baselines, CCA mainly incurs full-dataset covariance estimation and matrix decomposition, while Corr-AE, DAVAE, and SDCMR rely on encoder–decoder reconstruction, with DAVAE and SDCMR further introducing variational or adversarial branches. ACMR, DDAC, and AAL require alternating adversarial updates, whereas DSCMR and C3CMR construct batchwise pair relations and therefore also contain $O(n^2d')$ terms. Graph-based methods such as DGMS and ICMR-DCT similarly incur graph-construction or graph-attention costs that scale with the number of sample relations. GCMR-IMA avoids backpropagation through the frozen CLIP encoder, but its graph construction, multi-head attention, and missing-modality transformation retain quadratic batch-level costs. Therefore, its complexity should be viewed as a controllable robustness–computation trade-off rather than an unconditional efficiency advantage over all baselines.

## Experiments

### Datasets and evaluation metrics

To systematically evaluate the effectiveness of the proposed method, extensive experiments are conducted on three publicly available benchmark datasets: Pascal Sentence, Wikipedia, and NUS-WIDE-10K. The detailed configurations of each dataset are as follows:

- *Pascal Sentence* consists of 1000 visual samples across 20 semantic categories. Each image is annotated with five semantically relevant textual descriptions. Following the standard data split protocol, 800 samples are used for training, and the remaining 200 samples are used for testing.
- *Wikipedia* comprises 2866 image-text pairs, with each pair collected from the Wikipedia online encyclopedia, ensuring rich semantic correlations. The experiments adopt the official data split, where 2173 samples form the training set and 462 samples are used for testing.
- *NUS-WIDE-10K*, a subset of the larger NUS-WIDE dataset, contains 10,000 image-text pairs spanning 10 semantic categories. The dataset is partitioned into 8000 training samples and 2000 testing samples to ensure the reliability and comparability of experimental results.

To fairly compare retrieval performance, we adopt the Mean Average Precision (mAP) as the evaluation metric, which is widely used in cross-modal retrieval tasks due to its ability to reflect both precision and ranking quality. For each query, the Average Precision (AP) is computed as:

28 $$\begin{aligned} \textrm{AP}(q) = \frac{1}{R_q} \sum _{k=1}^{N} P(k) \cdot \delta (k), \end{aligned}$$

where $R_q$ is the number of relevant items for query *q*, *P*(*k*) is the precision at the top-*k* retrieved results, and *δ (k) = 1* if the *k*-th retrieved item is relevant, otherwise 0. Then, the mAP over all queries is calculated as:

29 $$\begin{aligned} \textrm{mAP} = \frac{1}{Q} \sum _{q=1}^{Q} \textrm{AP}(q), \end{aligned}$$

where *Q* is the total number of queries, all reported results include mAP values for both retrieval directions, Image-to-Text (I2T) and Text-to-Image (T2I), as well as their averages, to evaluate cross-modal alignment comprehensively.

### Baselines

For comprehensive evaluation, we compare GCMR-IMA with the following representative cross-modal retrieval methods:

1. *CCA*^2^: Employs kernel canonical correlation analysis to learn joint semantic representations of web images and texts for cross-modal comparison.
2. *Corr-AE*^7^: Introduces correspondence autoencoders that align uni-modal hidden features to learn modality-invariant representations.
3. *Deep-SM*^8^: Leverages CNNs with pretrained ImageNet features and fine-tuning, combined with deep semantic matching for multi-label alignment.
4. *CMDN*^28^: Proposes a hierarchical model capturing intra- and inter-modal correlations via a two-stage deep network.
5. *ACMR*^29^: Constructs a modality-invariant subspace through adversarial training with triplet constraints and a feature-projector–modality-classifier interplay.
6. *DSCMR*^9^: Jointly optimizes discriminative and modality-invariant representations via deep supervision.
7. *DAVAE*^11^: Using VGG-19 visual features, DAVAE learns dual-aligned variational representations and reconstructs missing modalities from Sentence-CNN or bag-of-words text features.
8. *C3CMR*^10^: Combines multi-level contrastive learning with VGG-19 image features to improve discriminability, while BERT supplies the textual representations.
9. *DDAC*^30^: Performs dual discriminant adversarial alignment between visual and textual embeddings, which are initialized from VGG-19 and a Word2Vec–Sentence-CNN pipeline, respectively.
10. *AAL*^31^: Built on image representations from VGG-19 and Word2Vec text embeddings, AAL adaptively balances adversarial objectives for cross-modal alignment.
11. *DGMS*^14^: Constructs dual graph-structured semantic subspaces, with VGG-19 and Text-CNN providing the image and text inputs, respectively.
12. *ICMR-DCT*^15^: With both modalities represented by pretrained CLIP, ICMR-DCT transfers deep correlations through graph-attention autoencoders.
13. *SDCMR*^12^: Separates shared from modality-specific semantics, using VGG-19 for visual encoding and WordCNN for textual representation.

In our implementation, we set the batch size to 100 with an initial learning rate of 0.0001 and a weight decay coefficient of 0.01. GCMR-IMA and its ablation variants are implemented in PyTorch and evaluated on a Linux server equipped with an NVIDIA A40 GPU.

Since most baseline results are taken from their original publications, the compared methods follow their respective feature extraction protocols, including recent approaches that also adopt CLIP representations. Therefore, the comparison reflects the reported end-to-end performance of each method rather than a strictly backbone-controlled evaluation.

### Complete cross-modal retrieval

To evaluate the performance of the proposed method under complete cross-modal retrieval scenarios, we conducted extensive experiments on three benchmark datasets: Pascal Sentence, Wikipedia, and NUS-WIDE-10K. A comprehensive set of baselines was selected, including both traditional statistical models (e.g. CCA, Corr-AE), early deep learning approaches (e.g. Deep-SM, CMDN), and a range of recent advanced methods such as ACMR, DSCMR, DAVAE, C3CMR, AAL, DDAC, and newly added models like DGMS, ICMR-DCT, and SDCMR. These models encompass a range of design paradigms, including dual-branch encoders, adversarial learning, dynamic graph structures, and transformer-based modality fusion. All methods were evaluated using mAP under both I2T and T2I retrieval settings.

As reported in Table 2, GCMR-IMA consistently achieves competitive and often best-performing results across all datasets. On the Pascal Sentence dataset, GCMR-IMA achieves an I2T mAP of 0.764 and a T2I mAP of 0.754, with an average mAP of 0.759, outperforming most previous baselines and closely approaching the best result from ICMR-DCT. On the Wikipedia dataset, GCMR-IMA achieves the highest average mAP of 0.603, slightly exceeding ICMR-DCT (0.602), and significantly outperforms all other methods, including SDCMR, which, although showing strong performance on image queries, suffers from weaker text retrieval. On the challenging and large-scale NUS-WIDE-10K dataset, GCMR-IMA achieves the best overall performance with an average mAP of 0.626, demonstrating robust generalization across both modalities.

**Table 2:** Cross-modal retrieval performance on three benchmark datasets.

Methods	Pascal sentence			Wikipedia			NUS-WIDE-10K		
	I2T	T2I	Avg.	I2T	T2I	Avg.	I2T	T2I	Avg.
CCA	0.225	0.227	0.226	0.258	0.250	0.254	0.258	0.250	0.254
Corr-AE	0.532	0.521	0.527	0.402	0.395	0.399	0.402	0.395	0.399
Deep-SM	0.560	0.539	0.550	0.458	0.345	0.402	0.389	0.496	0.443
CMDN	0.544	0.526	0.535	0.487	0.427	0.457	0.492	0.515	0.504
ACMR	0.671	0.676	0.673	0.511	0.467	0.489	0.544	0.538	0.541
DSCMR	0.710	0.722	0.716	0.521	0.479	0.500	0.552	0.542	0.547
DAVAE	0.713	0.708	0.710	0.586	0.564	0.578	0.606	0.582	0.594
C3CMR	0.702	0.715	0.708	0.553	0.483	0.518	0.573	0.589	0.581
AAL	0.712	0.719	0.716	0.541	0.480	0.510	–	–	–
DDAC	0.735	0.733	0.734	0.539	0.487	0.513	0.586	0.593	0.590
DGMS	*0.764*	0.730	0.747	0.558	0.494	0.526	0.601	0.609	0.605
ICMR-DCT	**0.767**	*0.753*	**0.760**	0.607	**0.596**	*0.602*	**0.624**	*0.626*	*0.625*
SDCMR	0.628	0.608	0.618	**0.683**	0.475	0.579	0.597	0.607	0.602
**GCMR-IMA**	0.764	**0.754**	*0.759*	0.613	*0.592*	**0.603**	*0.623*	**0.628**	**0.626**

Here, ’-’ indicates that the original value was not provided by the method. Significant values are in bold and italic.

Compared with recent strong competitors such as ICMR-DCT and DGMS, GCMR-IMA shows advantages in both semantic alignment and robustness under complex scenarios. It can be attributed to GCMR-IMA’s design, which combines intra-modal graph construction, inter-modal transformation, and a hierarchical alignment strategy. These improvements enable the model to capture structural correspondences and latent semantics across modalities with greater precision. Overall, the results validate GCMR-IMA as a strong and adaptable framework for cross-modal retrieval in both moderate and large-scale settings.

### Incomplete cross-modal retrieval

This section presents experiments under missing modalities, simulating real-world scenarios with limited paired data by evaluating six incomplete settings: (1) 50% paired data, 25% image-only data, and 25% text-only data; (2) 50% paired data, no image-only data, and 50% text-only data; (3) 50% paired data, 50% image-only data, and no text-only data; (4) 40% paired data, 30% image-only data, and 30% text-only data; (5) 30% paired data, 35% image-only data, and 35% text-only data; (6) 10% paired data, 45% image-only data, and 45% text-only data. As shown in Table 3.

**Table 3:** Missing-modality retrieval mAP under different missing rates on the three datasets.

Imbalance setting	Pascal sentence		Wikipedia		NUS-WIDE-10K	
	DAVAE	GCMR-IMA	DAVAE	GCMR-IMA	DAVAE	GCMR-IMA
(100%P, 0%I, 0%T)	0.709	*0.745*	0.574	**0.602**	0.592	**0.618**
(50%P, 25%I, 25%T)	**0.726**	0.728	0.571	*0.584*	0.587	0.611
(50%P, 0%I, 50%T)	0.643	0.734	*0.580*	0.581	*0.588*	0.608
(50%P, 50%I, 0%T)	0.707	**0.748**	0.555	0.572	0.585	*0.612*
(40%P, 30%I, 30%T)	*0.718*	0.719	0.570	0.577	0.577	0.591
(30%P, 35%I, 35%T)	0.710	0.717	**0.583**	0.582	**0.598**	0.608
(10%P, 45%I, 45%T)	0.630	0.711	0.547	0.577	0.579	0.592

Significant values are in bold and italic.

Comparative experiments with existing incomplete cross-modal retrieval methods, such as DAVAE, demonstrate that GCMR-IMA achieves significantly superior performance by constructing a dual-embedding joint graph structure to enable effective modality conversion, thereby uncovering inherent cross-modal correlations and generating high-quality missing modality representations. The core innovation of GCMR-IMA lies in its hierarchical design, which enables a GCN module to capture high-dimensional topological dependencies between modalities in a dynamic manner. At the same time, multi-head attention focuses on discriminative local features.

As evidenced by the results in Table 3, GCMR-IMA consistently outperforms DAVAE across all tested datasets and missing-modality scenarios. For example, under the (100%P, 0%I, 0%T) setting, GCMR-IMA improves mAP by 3.6–4.8% absolute (0.745 vs. 0.709, 0.602 vs. 0.574, and 0.618 vs. 0.592, respectively), with even larger gains in challenging imbalanced cases like (50%P, 50%I, 0%T) (0.748 vs. 0.707 on Pascal Sentence) and (10%P, 45%I, 45%T) (0.711 vs. 0.630).

This robustness stems from GCMR-IMA’s hierarchical semantic alignment mechanism . At high missing rates, the reduced number of reliable semantic anchors may increase the uncertainty of adjacency estimation and graph propagation. Modality-aware initialization, top-*k* sparsification, self-loop preservation, and the joint constraints imposed by $L_{\text {sim}}$ and $L_{\text {recon}}$ help restrict the propagation of unreliable edges while preserving node-specific information. The results therefore exhibit relatively smooth and controlled performance degradation as modality incompleteness increases.

### Ablation study

To further validate the effectiveness of the proposed method, we conducted a series of ablation studies focusing on the contributions of individual modules and loss components. The experiments are divided into four variants, each removing a specific loss term to examine its impact on model performance. Specifically: (a) without $L_{con}$, (b) without $L_{class}$, (c) without $L_{sim}$, and (d) without $L_{recon}$. The complete model, including all losses, is denoted as (e). Experimental results are summarized in Table 4.

**Table 4:** Sensitivity results of the loss function on the baseline datasets.

Methods	Wikipedia			Pascal sentence			NUS-WIDE-10K		
	I2T	T2I	Avg.	I2T	T2I	Avg.	I2T	T2I	Avg.
(a)	0.572	0.578	0.575	0.756	0.727	0.742	0.589	0.591	0.590
(b)	0.594	0.578	0.586	0.743	0.731	0.737	0.608	0.595	0.602
(c)	0.582	0.589	0.586	0.758	0.729	0.744	0.593	0.589	0.591
(d)	*0.597*	0.587	*0.592*	*0.759*	0.736	*0.748*	*0.618*	0.615	*0.617*
(e)	**0.612**	**0.591**	**0.602**	**0.764**	**0.748**	**0.756**	**0.623**	**0.629**	**0.626**

Significant values are in bold and italic.

As shown in Table 4, the full GCMR-IMA model (e) achieves the best performance across all evaluation metrics–including I2T, T2I, and Average–on the Wikipedia, Pascal Sentence, and NUS-WIDE-10K datasets, with average mAPs of 0.602, 0.756, and 0.626, respectively. When the contrastive loss $L_{con}$ is removed (variant a), the average mAP on Wikipedia drops from 0.602 to 0.575, and similar declines are observed on Pascal Sentence (from 0.756 to 0.742) and NUS-WIDE-10K (from 0.626 to 0.590). Removing the classification loss $L_{class}$ (variant b) leads to a decrease of 0.016 on Wikipedia and 0.024 on NUS-WIDE-10K. The similarity loss $L_{sim}$ (variant c) also proves essential, particularly for the Pascal Sentence dataset, where the average mAP decreases from 0.756 to 0.744. Furthermore, the absence of reconstruction loss $L_{recon}$ (variant d) results in moderate but consistent performance degradation across all datasets.

To further evaluate the model’s robustness under incomplete cross-modal scenarios, we conducted additional ablation studies on the Pascal Sentence dataset under two partial supervision settings. The corresponding results are summarized in Table 5.

**Table 5:** Sensitivity results of the loss function under different missing rates.

Methods	50%P, 25%I, 25%T			10%P, 45%I, 45%T		
	I2T	T2I	Avg.	I2T	T2I	Avg.
(a)	0.724	0.702	0.713	0.688	0.674	0.681
(b)	0.718	0.709	0.714	0.689	0.685	0.687
(c)	0.728	0.715	0.722	0.687	0.682	0.685
(d)	*0.736*	*0.722*	*0.729*	*0.701*	*0.696*	*0.699*
(e)	**0.743**	**0.731**	**0.737**	**0.721**	**0.712**	**0.717**

Significant values are in bold and italic.

As shown in Table 5, the full model (e) consistently achieves the highest retrieval performance across both partial settings, outperforming all ablated variants (a–d) on both I2T and T2I tasks. Notably, under the more challenging 10% pairing condition, the average mAP of the full model reaches 0.717–substantially higher than all counterparts–demonstrating its superior resilience to severe modality incompleteness. These results confirm the necessity of each loss component in preserving retrieval accuracy and further emphasize the adaptability and generalizability of the proposed loss framework across varying levels of supervision.

###  Fixed-coefficient sensitivity analysis

**Fig. 2:**
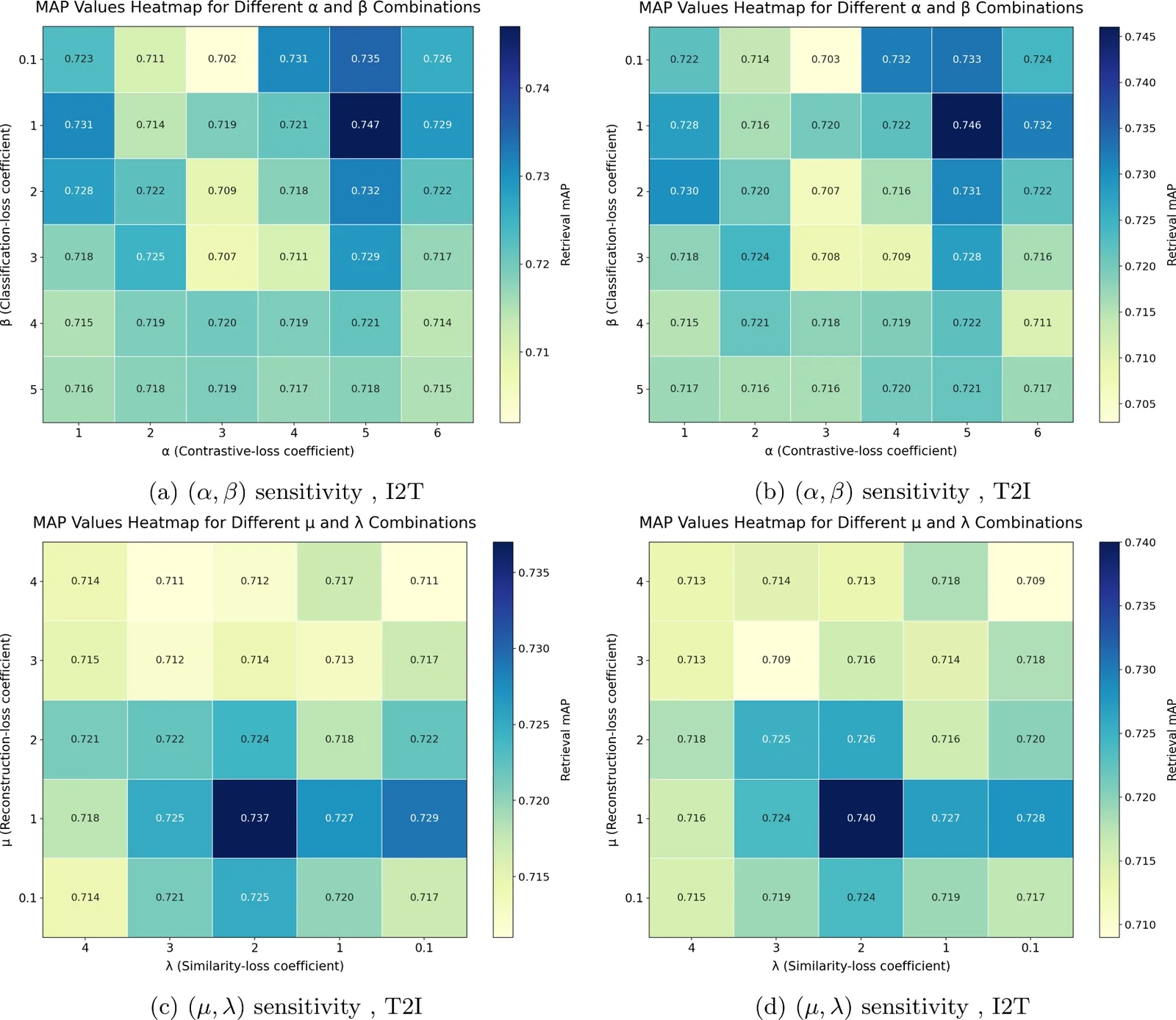
Fixed-coefficient sensitivity analysis under different loss-weight combinations. In Set 1, the horizontal and vertical axes denote *α* (the coefficient of $L_{\textrm{con}}$) and *β* (the coefficient of $L_{\textrm{class}}$), respectively. In Set 2, they denote *λ* (the coefficient of $L_{\textrm{sim}}$) and *μ* (the coefficient of $L_{\textrm{recon}}$), respectively. The color scale represents retrieval mAP, and each subfigure indicates the corresponding I2T or T2I retrieval direction.

The main experiments use the uncertainty-based weighting formulation in Eq. (25), where the learnable variables are the log-variance parameters $s_r$. Figure 2 provides an auxiliary fixed-coefficient sensitivity analysis: different manually specified combinations of $(\alpha ,\beta )$ and $(\mu ,\lambda )$ are evaluated only to examine the stability of the model under different relative loss scales. In Set 1, the horizontal axis denotes *α*, the coefficient of $L_{con}$, and the vertical axis denotes *β*, the coefficient of $L_{class}$. In Set 2, the horizontal axis denotes *λ*, the coefficient of $L_{sim}$, and the vertical axis denotes *μ*, the coefficient of $L_{recon}$. These coefficients are used only in the auxiliary sensitivity analysis, rather than as the learnable variables in the main experiments. The color scale represents retrieval mAP.

We examined two distinct sets of hyperparameter combinations: $(\alpha , \beta )$ and $(\mu , \lambda )$, and visualized their interactive effects on retrieval performance. As shown in Fig. 2b, the mAP achieves a relatively high value when *α = 5* and *β = 1*, indicating peak performance in the corresponding heatmap. Similarly, Fig. 2dshows that retrieval performance is optimized when *μ = 1* and *λ = 2*.

These heatmaps should not be interpreted as optimization trajectories of the learnable $s_r$. Instead, they show that the model maintains competitive performance across a range of fixed coefficient combinations, while the main experiments rely on the uncertainty-based weighting mechanism in Eq. (25).

### Precision-recall curve evaluation

In scenarios where the data distribution is imbalanced or the emphasis lies on accurately retrieving positive instances–such as in cross-modal retrieval tasks–the traditional accuracy or even ROC-based metrics may fall short in effectively capturing model performance. To address this limitation, we adopt the Precision-Recall (PR) curve as an auxiliary yet insightful evaluation tool. The PR curve provides a more informative assessment in cases where the number of relevant (positive) samples is significantly smaller than the number of irrelevant ones, which is common in real-world retrieval settings.

**Fig. 3:**
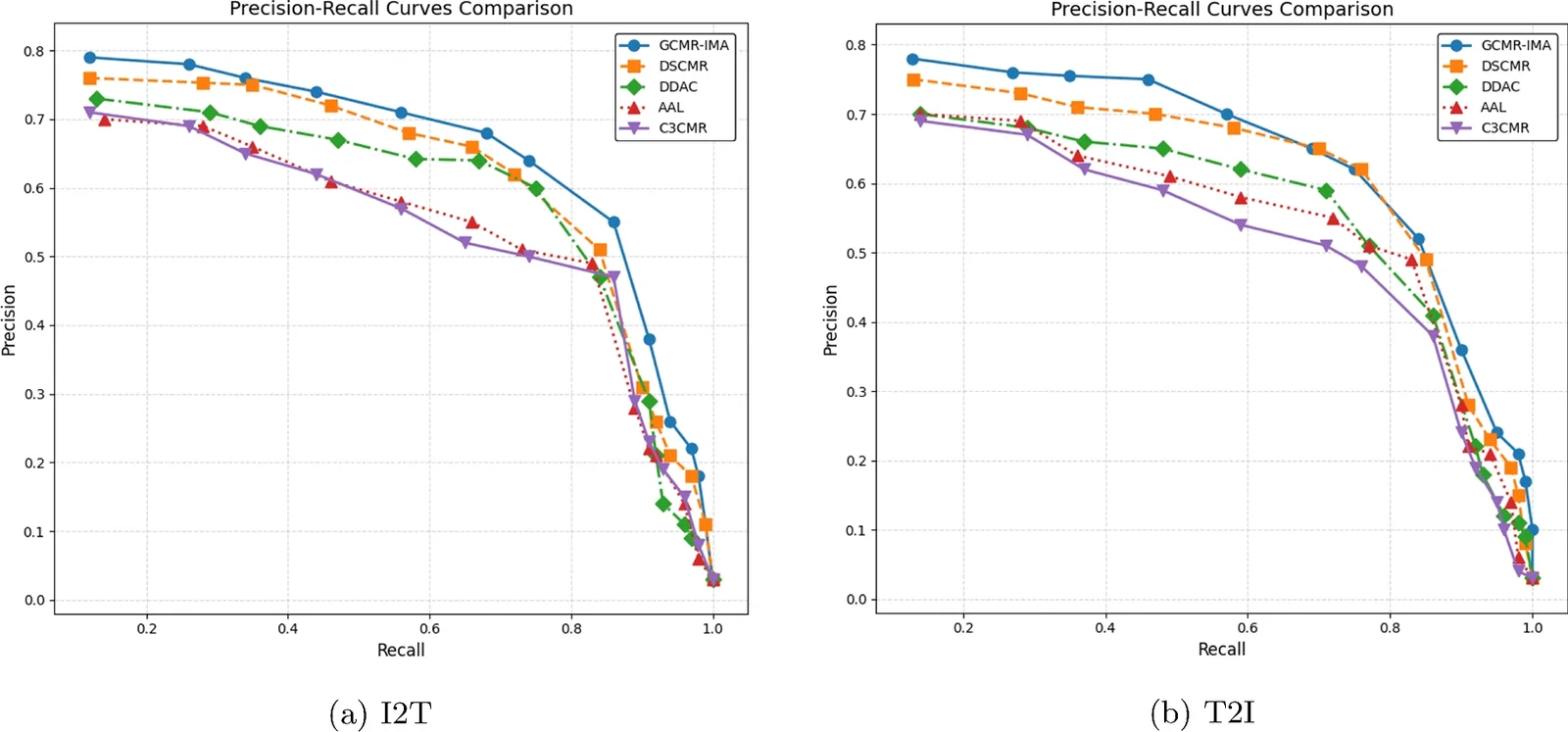
Precision-Recall curves of different baseline methods on the Pascal Sentence dataset. A larger area under the curve corresponds to superior retrieval performance.

As shown in Fig. 3, we illustrate the PR curves for five representative methods–GCMR-IMA, DSCMR, DDAC, AAL, and C3CMR–on the Pascal Sentence dataset, for both I2T and T2I retrieval tasks. It is well established that the Area Under the PR Curve (AUPRC) serves as a reliable indicator of model effectiveness, where a larger area implies better performance in distinguishing relevant from irrelevant matches.

Among the compared approaches, GCMR-IMA consistently demonstrates superior performance, especially in the high-recall region, where maintaining high precision becomes increasingly challenging. Its PR curve declines more gradually, indicating a more stable precision as recall increases. In contrast, the PR curves of DDAC, AAL, and C3CMR exhibit steeper drops and greater fluctuation, with precision values intersecting at varying recall levels. These patterns suggest that although the latter methods may achieve competitive performance under certain thresholds, they are generally less robust across the full recall spectrum.

### Feature visualization

**Fig. 4:**
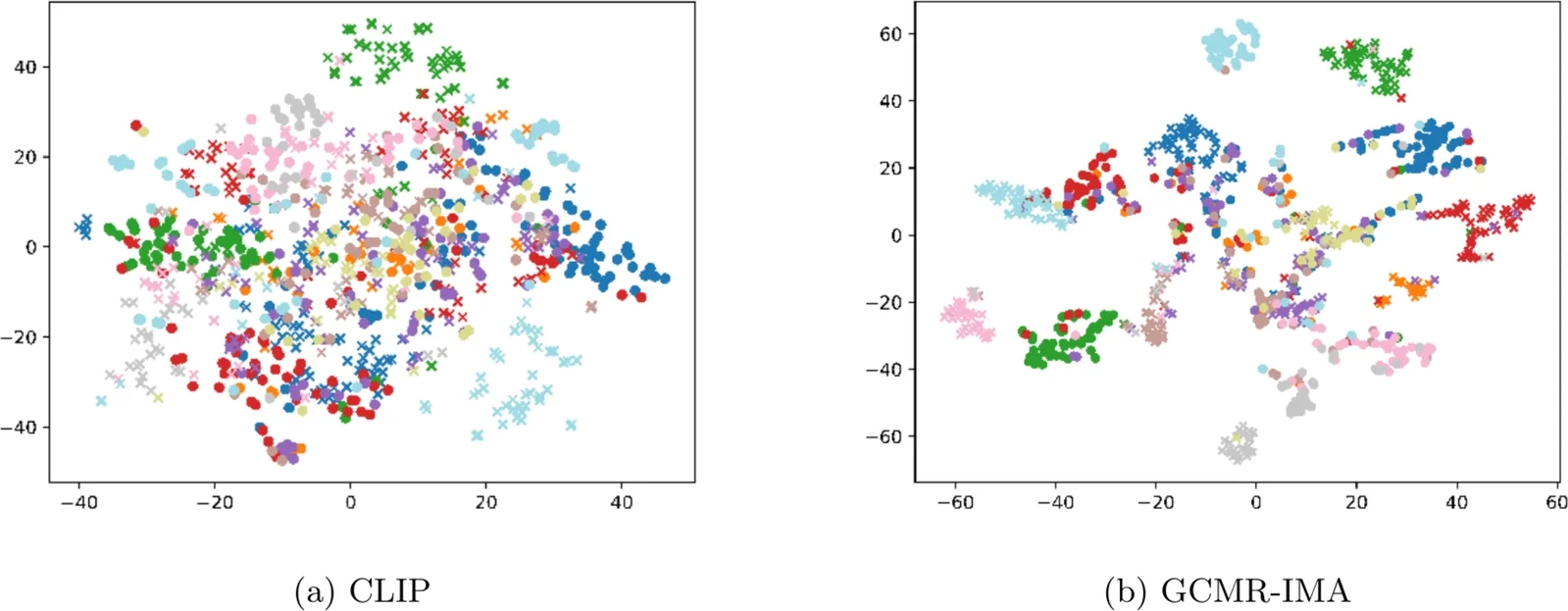
The t-SNE visualization of pure CLIP and GCMR-IMA representations on the Wikipedia dataset.

T-SNE is a commonly used method for visualizing high-dimensional data, capable of projecting features into two- or three-dimensional space for intuitive analysis. Figure 4 presents the visualization results on the Wikipedia dataset using features extracted by CLIP and GCMR-IMA. The three subfigures on the left display the image features, text features, and their joint distribution, as produced by CLIP, with different colors indicating distinct categories. The right three subfigures display the corresponding results generated by GCMR-IMA.

In the CLIP visualizations, data points appear more scattered and overlapping across categories, reflecting that while CLIP captures diverse features, the overall structure is relatively loose and lacks clear semantic clustering. It suggests that despite CLIP’s strong performance in feature extraction, the resulting representations remain entangled in the shared space, which limits their direct applicability to retrieval tasks. In contrast, GCMR-IMA produces more structured feature distributions. As shown on the right side, data points begin to form tighter clusters by category, especially within the image space, indicating a stronger ability to aggregate semantically similar features. This enhanced semantic coherence is critical for improving the accuracy and efficiency of cross-modal retrieval.

The strength of GCMR-IMA lies in its ability to leverage CLIP’s initial feature representations as a foundation, while further refining them through intra-modal graph construction and cross-modal semantic alignment. By incorporating graph convolution and attention mechanisms, GCMR-IMA captures hierarchical semantic information, significantly improving the organization of the feature space. These visualizations confirm that the proposed fusion strategy yields more orderly and discriminative feature distributions, which are crucial for achieving precise cross-modal matching.

**Fig. 5:**
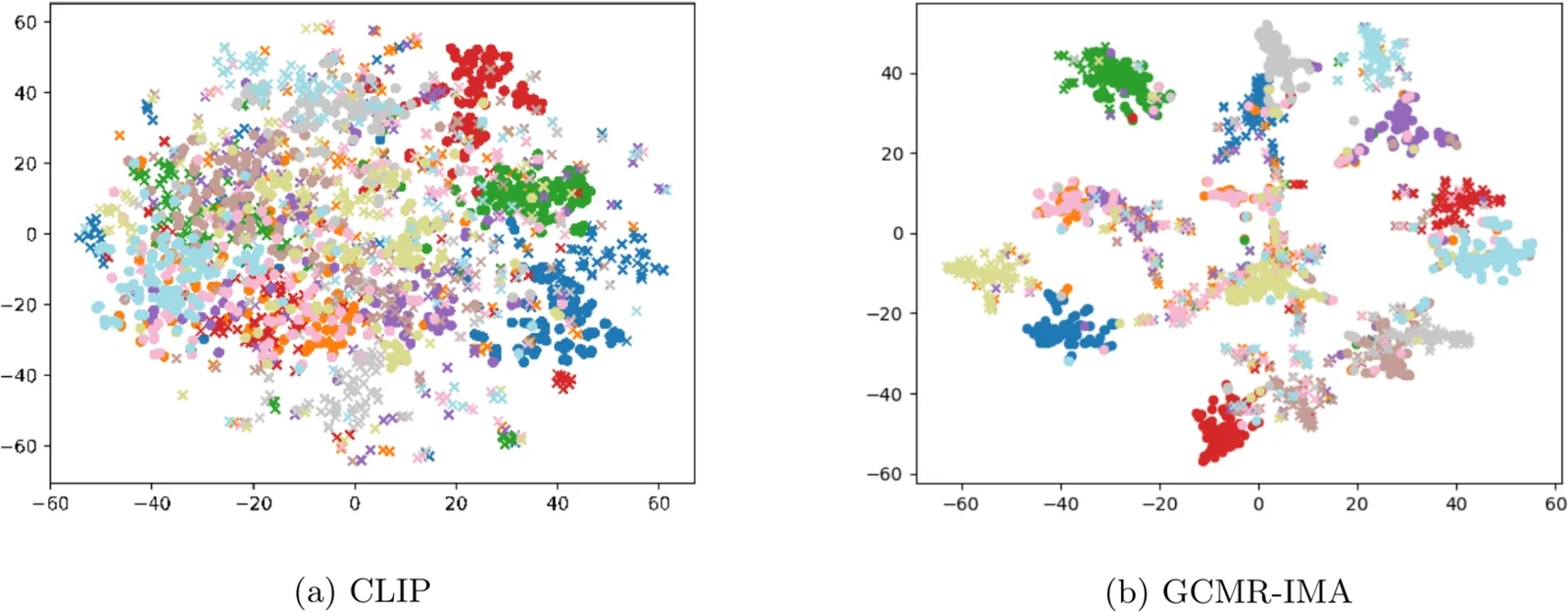
The t-SNE visualization of pure CLIP and GCMR-IMA representations on the NUS-WIDE-10K dataset.

Similarly, the visualization on the NUS-WIDE-10K dataset (Fig. 5) reveals patterns both consistent with previous datasets and unique to its structure. In the left subfigure, the data points are widely scattered with overlapping colors, indicating substantial inter-class confusion in the original feature space. In contrast, the right subfigure shows a tendency toward clustering. Although less pronounced than in some previous models, this shift suggests that the GCMR-IMA model has partially extracted and organized feature representations, enabling semantically similar instances to become more spatially coherent, which benefits the subsequent cross-modal retrieval performance.

## Conclusion

This work proposed GCMR-IMA, a graph-based framework for incomplete image–text retrieval. It combines missing-aware embedding preprocessing, sparse semantic graph learning, graph convolution, multi-head attention, and graph-guided cross-modal transformation to model global semantic dependencies and refine missing-modality representations. Experiments on Pascal Sentence, Wikipedia, and NUS-WIDE-10K demonstrate competitive performance under both complete and incomplete settings, showing that GCMR-IMA improves graph reliability and semantic consistency when one modality is unavailable.

The current framework is designed primarily for static image–text pairs. It does not explicitly model temporal evolution, asynchronous observations, or higher-order interactions among multiple segments or modalities. Its graph quality also depends on the reliability of observed features and semantic neighbors, so extremely high missing rates or severe noise may still introduce inaccurate edges. Moreover, pairwise graph construction and global attention contain quadratic computational terms, which may limit their direct application to long videos or large-scale multimodal sequences.

The framework can, in principle, be extended to video–text retrieval and audio–visual understanding by replacing the image and text encoders with task-specific video, audio, and language encoders, and by treating frames or temporal segments as graph nodes. Temporal or dynamic graphs could then capture evolving dependencies, while heterogeneous graphs or hypergraphs could represent asynchronous and higher-order multimodal relations. However, such extensions require more reliable temporal alignment, efficient long-sequence graph construction, dynamic neighbor updating, and robust propagation under missing or noisy segments. Future work will therefore investigate lightweight dynamic graphs, hierarchical temporal aggregation, and inductive graph learning to improve scalability and transferability to broader multimodal tasks.

## Data Availability

Data will be made available on request.
